# PROTOCOL: Mass deworming for soil‐transmitted helminths and schistosomiasis among pregnant women: a systematic review and individual participant data meta‐analysis

**DOI:** 10.1002/CL2.207

**Published:** 2018-06-12

**Authors:** Rehana A Salam, Philippa Middleton, Maria Makrides, Vivian Welch, Michelle Gaffey, Simon Cousens, Zulfiqar Bhutta

## Background

### The problem, condition or issue

Soil transmitted helminthiasis (STH) are a group of diseases caused by infection with four intestinal parasites: *Ascaris lumbricoides* (roundworm), *Trichuris trichiura* (whip worm), *Necator americanus* (hookworm) and *Ancylostoma duodenale* (hookworm). Schistosomiasis is also a parasitic disease caused by blood flukes of the genus *Schistosoma*. Six species of schistosomes are responsible for infection in humans: *Schistosoma guineensis*, *S.haematobium, S. intercalatum, S. japonicum, S. mansoni and S. mekongi; S. haematobium* and *S. mansoni* are predominant causes of disease. An estimated 438.9 million people were infected with hookworm in 2010, 819.0 million with roundworms and 464.6 million with whipworm. STH altogether, contributed to a total of 4.98 million years lived with disability (YLDs) (Pullan, Smith, Jasrasaria, & Brooker, 2014). Of these YLDs, 65 per cent were attributable to hookworm, 22 per cent to roundworm and the remaining 13 per cent to whipworm. In terms of geographical distribution, around 67 per cent of STH occurred in Asia contributing to 68 per cent of the YLDs (Pullan et al., 2014). Over 270 million preschool‐age children and over 600 million school‐age children live in STH endemic areas and an estimated four million pregnancies a year are complicated by maternal hookworm infection alone (Bundy, Chan, & Savioli, 1995; WHO, 2005).

Anaemia is one of the most common side effects of infection with STH or *schistosomes,* due to blood loss in the intestine or urinary tract. Women in low‐middle‐income countries (LMICs) are especially prone since they may be pregnant or lactating for as much as half of their reproductive lives with over 50 per cent of the pregnant women having iron‐deficiency anaemia. Although iron‐deficiency anaemia is multifactorial, hookworm infection is an important contributory factor in endemic areas, especially among women of reproductive age. An analysis on anaemia epidemiology based on data from the Global Burden of Diseases, Injuries and Risk Factors (GBD) 2010 Study suggested that hookworm and Schistosomiasis were among the top ten causes of anaemia among females in 2010 (Kassebaum et al., 2014). It is the leading cause of pathological blood loss in tropical and subtropical regions (Pawlowski, Schad, & Stott, 1991). Moreover there is a direct association between the intensity of STH infection, blood loss and consequent anaemia, especially for hookworms (Bundy et al., 1995; Chan, Medley, Jamison, & Bundy, 1994; Larocque, Casapia, Gotuzzo, & GYORKOS, 2005). The association between anaemia during pregnancy and adverse pregnancy outcomes, including low birth weight (LBW), preterm birth, perinatal mortality and infant survival have already been documented (Rahman et al., 2016; Sifakis & Pharmakides, 2000). Furthermore, the chances of favourable pregnancy outcomes are reduced by 30 per cent to 45 per cent in anaemic mothers, with their infants having less than one half of normal iron reserves (Rahman et al., 2016).

Mass deworming (treatment at a large scale irrespective of the diseases status) along with the water, sanitation and hygiene (WASH) interventions are generally accepted as effective measures to prevent and treat STH and Schistosomiasis. However, findings from existing studies are conflicting and the effectiveness of mass deworming in improving various maternal and child health outcomes is a current source of debate (Turner et al., 2015). Critical appraisal of existing studies suggests that these fail to account for various factors that could modify the effectiveness of deworming including nutritional status, type of infection, worm burden and concomitant interventions (Barry, Simon, Mistry, & Hotez, 2013; Turner et al., 2015)

### The intervention

The World Health Organization (WHO) recommends mass deworming for STH and Schistosomiasis depending on prevalence of worm infection. Preventive chemotherapy (deworming), using single‐dose albendazole (400 mg) or mebendazole (500 mg), is recommended as a public health intervention for pregnant women, after the first trimester, living in areas where both: (i) the baseline prevalence of hookworm and/or *T. trichiura* infection is 20 per cent or higher among pregnant women, and (ii) anaemia is a severe public health problem, with a prevalence of 40 per cent or higher among pregnant women, in order to reduce the worm burden of hookworm and *T. trichiura* infection (WHO, 2017). For Schistosomiasis, annual treatment with praziquantel in high risk communities (>50%) and once every two years in medium risk (>10% and <50%) is recommended and women can be treated with praziquantel at any stage of pregnancy and lactation (WHO, 2006). In addition to deworming; education on health and hygiene and provision of adequate sanitation is also recommended.

### How the intervention might work

STH and Schistosomiasis are a major public health concern since these parasites feed on blood and affect the supply of nutrients necessary for erythropoiesis; hence contributing to anaemia (Hotez & Cerami, 1983; Torlesse & Hodges, 2000). Additionally, STH may also lead to haemorrhage by releasing anticoagulant compounds, thereby leading to iron‐deficiency anaemia. Infection during pregnancy leads to an added demand for nutrients that are critical for foetal growth and development (Abrams & Miller, 2011; Blackwell, Snodgrass, Madimenos, & Sugiyama, 2010). Hookworms, in particular, along with other STH and *schistosomes* have been associated with reductions in haemoglobin and iron deficiency during pregnancy (Gyorkos, Gilbert, Larocque, & Casapía, 2011; Larocque et al., 2005; Muhangi et al., 2007; Ndyomugyenyi, Kabatereine, Olsen, & Magnussen, 2008; Nurdia, Sumarni, Hakim, & Winkvist, 2001). Additionally, STH and Schistosomiasis often occur with co‐infections in areas where malnutrition is already prevalent (Martin, Blackwell, Gurven, & Kaplan, 2013).

Deworming is regarded as the most effective means of controlling mortality and morbidity with STH and Schistosomiasis (WHO, 2006, 2017). Preventive chemotherapy (either alone or in combination) has been used as a public heath tool for preventing morbidity due to infection usually with more than one helminth at a time since many of the antihelminthic drugs are broad spectrum. In 1994, the WHO convened an informal consultation on hookworm infection and anaemia in girls and women, which promoted the use of antihelminthics in pregnancy after the first trimester in areas where these infections are endemic and where anaemia is prevalent, but it also recommended evaluation of the long‐term safety, particularly in terms of birth outcomes. Women can be treated with praziquantel for schistosomiasis at any stage of pregnancy and during lactation. Deworming during pregnancy is often accompanied with iron supplementation to reduce anaemia.

**Figure 1:**
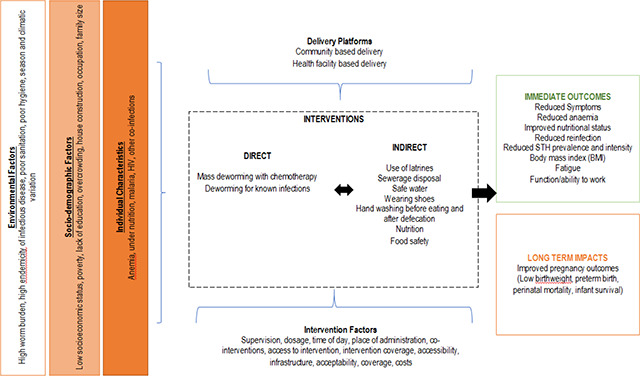
Logic model

There are various factors that could potentially modify the effectiveness of deworming including baseline nutritional status (anaemia and body mass index (BMI)), type of STH infection, treatment protocol, worm burden (particularly intensity of infection) and concomitant interventions (such as iron supplementation and other drugs such as praziquantel for Schistosomiasis). However, given the limited number of studies assessing the impact of deworming on maternal and newborn health outcomes (Salam, Haider, Humayun, & Bhutta, 2015a) and complex interactions helminths can have on maternal and infant immune function, health, and co‐infection risks (Blackwell, 2016), it is difficult to ascertain how these factors interplay. Currently, it is difficult to establish whether deworming during pregnancy has beneficial effects under certain conditions and limited effects under others and there exists a possibility that it is only beneficial in women with very high parasite burdens, dietary insufficiencies, or both (Blackwell, 2016). Moreover, all intestinal worms are not the same; not all intestinal worms respond to the same deworming medication; and not all infested individuals exhibit the disease. Reinfection depends on the prevalence and intensity of infection as well as environmental factors such as the WASH practices in the community. Figure 1 highlights the logic model.

### Why it is important to do the review

A Cochrane review of deworming in the second trimester of pregnancy including four trials and 4265 participants concluded that there was insufficient evidence to recommend deworming in pregnancy (Salam, Haider, Humayun, & Bhutta, 2015b). There was no impact of single dose of antihelminthic in the second trimester of pregnancy on maternal anaemia in the third trimester, low birth weight, preterm birth and perinatal mortality. A recent Campbell systematic review and network meta‐analysis with 47 randomised trials and >1 million children, found little to no overall effect on growth, attention and school attendance (Welch et al., 2016). However, these reviews were conducted at the study level, rather than using data for each individual participant, which limits the power to detect effect modification by individual participant characteristics that could potentially modify the effect of deworming including baseline nutritional status, type of STH infection, treatment protocol, worm burden and concomitant interventions (such as iron supplementation) (Barry et al., 2013; Turner et al., 2015).

Individual participant data (IPD) meta‐analysis refers to analysing data for each participant in the existing studies (Tierney, Pignon, et al., 2015; Tierney, Vale, et al., 2015). The term IPD refers to analysing data recorded for each participant in contrast to the aggregate study data in meta‐analysis. The advantage of an IPD analysis over aggregate meta‐analysis is that it has the potential to improve the quality of both the data and the analyses and consequently the reliability of the results (Tierney, Vale, et al., 2015). Furthermore, it also provides an opportunity to re‐analyse the data for the range of other possibilities for example, investigating the treatment effects varying by participant characteristics which is not possible with the aggregate data (Riley, Lambert, & Abo‐Zaid, 2010). An IPD approach will allow the evaluation of variation in effect estimates by various individual, socio‐demographic and environmental factors in pregnant women that could potentially modify the effectiveness of deworming during pregnancy.

Despite the availability of more recent global estimates on the burden and interventions for STH and Schistosomiasis, additional research is needed to understand the factors that explain the variation in the effect estimates of recommended interventions to prevent transmission. Existing studies fail to account for various factors that modify the effectiveness of deworming including underlying host and environment factors. IPD meta‐analysis would explore the question of whether mass deworming during pregnancy is more effective for subgroups of women defined by characteristics such as nutrition status and infection intensity. This understanding could help to develop targeted strategies to reach pregnant women with deworming and guide policy regarding deworming.

## Objectives

The objective of the review is to use IPD meta‐analysis to explore whether the effect of deworming among pregnant women on maternal and birth outcomes vary with individual characteristics (nutritional status, anaemia), intensity of infection (as assessed by egg count), infection status (including species of worm), socioeconomic status, sanitation environment and co‐interventions.

## Methodology

### Criteria for including and excluding studies

#### Types of study designs

We will include individually randomised controlled trials; cluster randomised controlled trials and quasi randomised studies since these would be the most appropriate design for the IPD meta‐analysis. No language or date restrictions will be applied.

#### Types of participants

Pregnant women receiving preventive or therapeutic deworming.

#### Types of interventions

Mass deworming using any drugs for STH and *Schistosomes* with or without co‐interventions such as food, micronutrients, iron or hygiene interventions compared with placebo, control, or other active interventions (e.g. iron, hygiene promotion) as comparators.

#### Types of outcome measures

Primary outcomes:

- Maternal anaemia at term (haemoglobin less than 11 g/dL)
- Maternal infection intensity (as reported by the study authors).

Secondary outcomes:

- Maternal haemoglobin at term
- Maternal ferritin
- Maternal anthropometric measures (height and weight)
- Maternal body mass index (BMI)
- Birth weight
- Low birth weight (LBW) (less than 2500 g)
- Preterm birth (birth before 37 weeks of gestation)
- Perinatal mortality (includes foetal death after 28 weeks of gestation and infant death that occurs at less than seven days of life)
- Stillbirth
- Congenital abnormalities
- Infant mortality.

We will not exclude on the basis of reported outcomes since some measured outcomes may not be reported in trial reports or abstracts.

#### Duration of follow‐up

We will not restrict inclusion based on the duration of follow‐up.

#### Types of settings

The settings will include any area where soil‐transmitted helminths or schistosomes are endemic. These could include studies conducted in either community settings or facility settings including hospitals, antenatal clinics, primary healthcare centres etc.

### Search strategy

We will search in the following databases: MEDLINE, CINAHL, LILACS, EMBASE, the Cochrane Library, Internet Documents in Economics Access Service (IDEAS), Google Scholar, Web of Sciences, Social Services Abstracts, WHO Global Health Library, Global Health CABI and CAB Abstracts. We will search grey literature in OpenGrey. We will search websites of relevant organizations such as the World Bank, World Food Program and International Food Policy Research Institute. We will also contact authors of studies and members of our advisory board for any unpublished studies or grey literature reporting eligible studies. We will check reference lists of relevant studies and reviews. We will also search for trials registered with ClinicalTrials.gov and the WHO International Clinical Trials Registry Platform (http://www.who.int/trialsearch/).

Titles and abstracts will be screened in duplicate by two reviewers. We will pilot‐test the screening criteria at both title and abstract screening stage and full text stage. We will use the PRISMA flow diagram to report eligibility of studies. We will retrieve full text of all studies which pass this first level screening. The full text review will also be done in duplicate by two reviewers, and agreement will be reached by consensus. Disagreements will be resolved by consultation with a third reviewer. No language or date limits will be applied. The tentative search strategy is attached as an appendix.

### Description of methods used in primary research

Randomised controlled trials of deworming include two‐arm trials as well as factorial trials, with women allocated either individually or by cluster‐randomization.

### Details of study coding categories and quality assessment

We will extract the study characteristics including details of the populations, setting, socio‐demographic characteristics, interventions, comparators, outcomes and study design in duplicate. Risk of bias will be assessed at the study as well as the outcome level. At the study level, two independent reviewers will perform quality appraisal for each study using the Cochrane risk of bias tool which assesses selection bias, performance bias, detection bias, attrition bias and reporting bias (Higgins, Altman, & Sterne, 2011). Disagreements will be resolved by discussion or consultation with a third reviewer. At the outcome level, we will summarise the quality of evidence according to the outcomes as per the Grading of Recommendations Assessment, Development and Evaluation (GRADE) criteria (Walker, Fischer‐Walker, Bryce, Bahl, & Cousens, 2010). A grade of “high”, “moderate”, “low” and “very low” will be used for grading the overall evidence indicating the strength of an effect on specific health outcome based on methodological flaws within the component studies, consistency of results across different studies, generalizability of research results to the wider patient base and how effective the treatments have been shown to be (Balshem et al., 2011). The two reviewers will discuss ratings and reach consensus. Disagreements will be resolved by consulting a third reviewer. We will develop a summary of findings table to show the effects for the primary outcomes of maternal anaemia and infection intensity; as well as the secondary outcomes of preterm birth, LBW and perinatal mortality since these outcomes assess long‐term effects, particularly in terms of birth outcomes.

### Statistical procedures and conventions

Trialists of the included trials will provide IPD by electronic transfer where possible or other means as needed. The individual trial data will be recoded as required and checked with respect to range, internal consistency, missing values, outliers, errors and consistency with published reports. Trial details such as randomization methods and intervention details will be cross‐checked against published reports, trial protocols and data collection sheets. Inconsistencies or missing data will be discussed with the individual trialists and attempts will be made to resolve any problems by consensus. We will not exclude any study based on the way the outcomes have been reported.

Data will be prepared into a flat spread‐sheet with the same fields for every study. We will consider the missing values for each variable as missing at random (MAR). We will use multiple imputation to impute the missing values for covariates at baseline (individual participant level variables) and outcome variables (primary and secondary outcomes). Imputation will be done using Proc MI in SAS/STAT (SAS Institute Inc., Cary, NC, USA). We will assess the robustness of the results by running a separate model excluding imputed data (i.e. complete case analysis). Studies with missing data on more than 50 per cent of outcome or covariate data will be included in the complete case analysis only. We will present both the base case and complete case analysis.

Descriptive characteristics of each study will be presented, with details on the participant characteristics, environment, worm species, prevalence, intensity of infection, geographic location, interventions, comparator, outcomes and risk of bias assessment. Following data items will be collected:

‐ Individual Level:
  - Infection intensity with *Ascaris, trichuris,* hookworm and schistosomes (across four levels of none, light, moderate and heavy, using the WHO cutoffs for each helminth, available at: http://apps.who.int/iris/bitstream/10665/44671/1/9789241548267_eng.pdf)
  - Anemia status (using WHO cutoffs by age and altitude of non‐anaemic, mild, moderate and severe, http://www.who.int/vmnis/indicators/haemoglobin.pdf)
  - Undernutrition (BMI<18.5 kg/m^2^)
  - Socioeconomic status (As defined by trial authors): We will assess whether the measurement of socioeconomic status can be compared across study settings and time.
  - Deworming drug used.
‐ Environmental Level:
  - WASH practices (As defined by trial authors)
  - Population level infection intensity (using WHO cut‐offs for each worm‐type, as above)

We will calculate the standardised difference between the published data and the IPD received from authors for baseline characteristics and baseline outcome assessment. For endline, we will replicate the effect measures reported in study publications and calculate the standardised difference between the IPD received and the study report (Austin, 2009). We will conduct a one‐stage IPD meta‐analysis using random‐effects multilevel meta‐regression models to examine the interactions between the covariates and the treatment. The comparison of interest for the pairwise analysis will include (but not restricted to) any deworming drug versus no deworming; one deworming drug versus other deworming drug or a combination of deworming drugs. We will construct forest plots for unadjusted direct treatment comparisons and adjusted treatment comparisons. We do not plan to conduct network meta‐analysis based on our previous experience with limited number of studies in the domain (Salam et al., 2015a). We will conduct pairwise analyses for each comparison of interest by entering all IPD data into a multilevel model, with each study as one cluster. We expect considerable heterogeneity between studies for each outcome; therefore, we will use a random effects model.

If IPD are not available for all trials, we will use a two‐part model with one part based on IPD data and the second part based on aggregate data from studies which do not provide IPD (Fisher, Copas, Tierney, & Parmar, 2011; Riley et al., 2008; Riley & Steyerberg, 2010). We will account for clustering as above by nesting clusters within studies. We will account for the pre‐defined covariates of infection intensity, baseline anaemia, baseline nutritional status, socioeconomic status and maternal education in the model.

### Measures of treatment effects

We will separately analyse the dichotomous and continuous outcomes. For dichotomous outcomes, we will present the results as summary risk ratios (RRs) with 95 per cent confidence intervals (CI). We will present continuous outcome data as either a mean difference (MD), if outcomes have been measured on the same scale, or a standardised mean difference (SMD), if outcomes have been measured on different scales, with 95 per cent CI.

### Assessment of clinical and methodological heterogeneity within treatment comparisons

Heterogeneity across trials in terms of subject characteristics, trial methodologies and treatment protocols will be assessed using visual plots, tables and homogeneity statistics. We will assess heterogeneity using visual inspection of forest plots for pairwise analyses as well as statistical tests of heterogeneity (I^2^). In addition to I^2^, we will also assess between‐study variance (variation across study findings beyond random sampling error) by the variance of the distribution of the true study effects, commonly denoted as τ2.

### Publication bias

A funnel plot will be plotted for comparisons and outcomes with >10 studies. We will use Egger's test for asymmetry and visual inspection to assess the presence of publication bias and/or selective reporting.

### Subgroup analyses

If sufficient data are available, sub‐group analyses will be conducted to assess effects across both individual‐level as well as environment‐level characteristics. We will compare the results of models with subgroup analyses by assessing the size of quantitative or qualitative differences in effects, the statistical significance of tests for interactions, assessing between‐study variance and assessing the goodness of fit of the models using the likelihood ratio. Before conducting subgroup analyses, we will assess the distribution of each variable. If there are insufficient participants in some categories, the levels may be combined. The following individual and environment level effect modifiers will be assessed:

‐ Individual Level:
  - Infection intensity with *Ascaris, trichuris,* hookworm and schistosomes (across four levels of none, light, moderate and heavy, using the WHO cutoffs for each helminth, available at: http://apps.who.int/iris/bitstream/10665/44671/1/9789241548267?eng.pdf)
  - Anemia status (using WHO cutoffs by age and altitude of non‐anaemic, mild, moderate and severe, http://www.who.int/vmnis/indicators/haemoglobin.pdf)
  - Undernutrition (BMI<18.5 kg/m^2^)
  - Socioeconomic status (As defined by trial authors): We will assess whether the measurement of socioeconomic status can be compared across study settings and time.
‐ Environmental Level:
  - WASH practices (As defined by trial authors)

Population level infection intensity (using WHO cut‐offs for each worm‐type, as above)

### Sensitivity analyses

If sufficient data are available, we will conduct sensitivity analyses to assess robustness of results when restricted to studies at low risk of bias for sequence generation, allocation concealment and blinding of participants. We will assess whether results are robust to excluding imputed data (i.e. complete case analysis).

### Data management

Data will be transferred to SAS as a common platform for all studies, using a common data dictionary. We will check IPD data for consistency immediately upon receiving datasets for outlier individuals (e.g. with duplicate participant IDs, unrealistic date ranges). We will compare the IPD from authors with the aggregate data reported in the articles. Any missing or unusual data will be flagged for discussion with the trial author or statistician. We will ask for clarification from the authors to establish reasons for the errors, and correct them if possible. Any requests for authors will be discussed when the data are provided, such as clarification of trial risk of bias, conduct or eligibility criteria. We will also run the same statistical analysis as the authors to check for consistency with the published paper (Stewart et al., 2015). We will request statements of ethics approval from each study and we will not include data from studies that did not receive ethics approval. We will request that all data be transferred without any identifiers.

### Treatment of qualitative research

We do not plan to include qualitative research.

## Review Authors

**Lead review author:** The lead author is the person who develops and co‐ordinates the review team, discusses and assigns roles for individual members of the review team, liaises with the editorial base and takes responsibility for the on‐going updates of the review.

**Table:** 

Name:	Rehana A Salam
Title:	Lecturer, PhD Candidate
Affiliation:	Healthy Mothers, Babies and Children, South Australian Health and Medical Research Institute, Adelaide, Australia and University of Adelaide, Adelaide, Australia
Address:	Women's and Children's Hospital, 72 King William Rd, North
City, State, Province or County:	Adelaide SA
Postal Code:	5006
Country:	Australia
Phone:	451321440
Email:	rehana.abdussalam@adelaide.edu.au
**Co‐authors:**	
Name:	Philippa Middleton
Title:	Dr. Associate Professor
Affiliation:	Healthy Mothers, Babies and Children, South Australian Health and Medical Research Institute, Adelaide, Australia and Robinson Research Institute, University of Adelaide, Adelaide, Australia
Address:	Women's and Children's Hospital, 72 King William Rd, North
City, State, Province or County:	Adelaide SA
Postal Code:	5006
Country:	Australia
Phone:	(08) 8161 7612
Email:	philippa.middleton@adelaide.edu.au
Name:	Maria Makrides
Title:	Professor
Affiliation:	Healthy Mothers, Babies and Children, South Australian Health and Medical Research Institute, Adelaide, Australia
Address:	Women's and Children's Hospital, 72 King William Rd, North
City, State, Province or County:	Adelaide SA
Postal Code:	5006
Country:	Australia
Phone:	(08) 8161 6067
Email:	Maria.Makrides@sahmri.com
Name:	Vivian Welch
Title:	Director, Methods Centre, Bruyère Research Institute; Assistant Professor
Affiliation:	School of Epidemiology, Public Health and Preventive Medicine, University of Ottawa
Address:	304b ‐ 85 Primrose Avenue,
City, State, Province or County:	Ottawa, Ontario
Postal Code:	K1R 6M1
Country:	Canada
Phone:	+1 613‐562‐6262 ext 2904
Email:	vivian.welch@uottawa.ca
Name:	Michelle Gaffey
Title:	Senior Research Manager
Affiliation:	Centre for Global Child Health, The Hospital for Sick Children
Address:	555 University Ave
City, State, Province or County:	Toronto, Ontario
Postal Code:	M5G 1X8
Country:	Canada
Phone:	+1 416 813 7654 x309108
Email:	michelle.gaffey@sickkids.ca
Name:	Simon Cousens
Title:	Professor
Affiliation:	London School of Hygiene and Tropical Medicine (LSHTM)
Address:	Room 116‐LSHTM Keppel Street
City, State, Province or County:	London
Postal Code:	WC1E 7HT
Country:	UK
Phone:	+44 (20) 7927 2422
Email:	simon.cousens@lshtm.ac.uk
Name:	Zulfiqar Bhutta
Title:	Co‐Director, Centre for Global Child Health; Professor
Affiliation:	The Hospital for Sick Children
Address:	555 University Avenue
City, State, Province or County:	Toronto, Ontario
Postal Code:	M5G 1X8
Country:	Canada
Phone:	416‐813‐7654 ext 328532
Email:	zulfiqar.bhutta@sickkids.ca

## Roles and responsibilities

Please give a brief description of content and methodological expertise within the review team. It is recommended to have at least one person on the review team who has content expertise, at least one person who has methodological expertise and at least one person who has statistical expertise. It is also recommended to have one person with information retrieval expertise. Please note that this is the *recommended optimal* review team composition.

- Content: Zulfiqar Bhutta, Rehana Salam, Michelle Gaffey, Philippa Middleton, and Maria Makrides, Robert Black and Celia Holland have maternal health and nutritional expertise.
- Systematic review methods: Rehana Salam, Philippa Middleton and Maria Makrides have methodological expertise.
- Statistical analysis: Simon Cousens has statistical expertise.
- Information retrieval: Rehana Salam and Michelle Gaffey have information retrieval expertise.

Advisory Group: Robert Black, Celia Holland, Deirdre Hollingsworth, Sue Horton, Sanjay Wijesekera

## Sources of support

This review is funded by the Bill and Melinda Gates Foundation (Funding reference number: OPP1140742). The Gates Foundation will have no influence on the conclusions or publication.

## Declarations of interest

Rehana Salam, Michelle Gaffey, Robert Black, Simon Cousens, Zulfiqar Bhutta, Celia Holland, Philippa Middleton, and Maria Makrides have no conflict of interest, financial or otherwise that may influence judgments made in this review.

Vivian Welch is Editor in Chief of Campbell.

## Preliminary timeframe

Approximate date for submission of the systematic review: September 2018

**Table:** 

	Feb	Mar	Apr	May	Jun	Jul	Aug	Sep
Protocol submission	X							
Searching and screening	X	X						
Data extraction		X	X					
Synthesis			X	X				
Interpretation and write up					X	X		
Publication							X	X

## Plans for updating the review

Rehana A Salam will be responsible for updating the review.

## AUTHOR DECLARATION

### Authors' responsibilities

By completing this form, you accept responsibility for preparing, maintaining and updating the review in accordance with Campbell Collaboration policy. The Campbell Collaboration will provide as much support as possible to assist with the preparation of the review.

A draft review must be submitted to the relevant Coordinating Group within two years of protocol publication. If drafts are not submitted before the agreed deadlines, or if we are unable to contact you for an extended period, the relevant Coordinating Group has the right to de‐register the title or transfer the title to alternative authors. The Coordinating Group also has the right to de‐register or transfer the title if it does not meet the standards of the Coordinating Group and/or the Campbell Collaboration.

You accept responsibility for maintaining the review in light of new evidence, comments and criticisms, and other developments, and updating the review at least once every five years, or, if requested, transferring responsibility for maintaining the review to others as agreed with the Coordinating Group.

### Publication in the Campbell Library

The support of the Coordinating Group in preparing your review is conditional upon your agreement to publish the protocol, finished review, and subsequent updates in the Campbell Library. The Campbell Collaboration places no restrictions on publication of the findings of a Campbell systematic review in a more abbreviated form as a journal article either before or after the publication of the monograph version in Campbell Systematic Reviews. Some journals, however, have restrictions that preclude publication of findings that have been, or will be, reported elsewhere and authors considering publication in such a journal should be aware of possible conflict with publication of the monograph version in Campbell Systematic Reviews. Publication in a journal after publication or in press status in Campbell Systematic Reviews should acknowledge the Campbell version and include a citation to it. Note that systematic reviews published in Campbell Systematic Reviews and co‐registered with the Cochrane Collaboration may have additional requirements or restrictions for co‐publication. Review authors accept responsibility for meeting any co‐publication requirements.

**I understand the commitment required to undertake a Campbell review, and agree to publish in the Campbell Library. Signed on behalf of the authors**:

**Table:** 

**Form completed by: Rehana A Salam**	**Date: 5 February 2018**

## Appendix

Database: Ovid MEDLINE(R) In‐Process & Other Non‐Indexed Citations and Ovid MEDLINE(R)

1 flukes.tw.

2 platyhelminth*.tw.

3 whipworm*.tw.

4 whip worm*.tw.

5 hookworm*.tw.

6 hookworm*.tw.

7 hook worm*.tw.

8 roundworm*.tw.

9 round worm*.tw.

10 geohelminth*.tw.

11 ancylostoma*.tw.

12 Necator*.tw.

13 Ascaris.tw.

14 Ascaridida.tw.

15 Ancylostoma.tw.

16 Necator americanus.tw.

17 Trichuris.tw.

18 Trichuroidea.tw.

19 Adenophorea.tw.

20 Enoplida.tw.

21 Ascaridida.tw.

22 Platyhelminth*.tw.

23 Rotifera.tw.

24 trichuriasis.tw.

25 ascariasis.tw.

26 ancylostomiasis.tw.

27 ascarid*.tw.

28 schistosom*.tw.

29 bilharziosis.tw.

30 bilharzia*.tw.

31 exp Schistosoma/

32 or/1‐31

33 Albendazole/

34 Mebendazole/

35 exp Piperazines/

36 Levamisole/

37 exp Pyrantel/

38 Ivermectin/

39 exp Anthelmintics/

40 Ivermectin.tw.

41 Albendazole.tw.

42 Mebendazole.tw.

43 Piperazine*.tw.

44 Levamisole.tw.

45 pyrantel.tw.

46 tiabendazole.tw.

47 anthelmint*.tw.

48 Anticestodal.tw.

49 Antiplatyhelmintic.tw.

50 Anti‐platyhelmintic.tw.

51 Albendazole.tw.

52 Dichlorophen.tw.

53 Niclosamide.tw.

54 Bithionol.tw.

55 Diamfenetide.tw.

56 Nitroxinil.tw.

57 Oxyclozanide.tw.

58 Rafoxanide.tw.

59 Schistosomicid*.tw.

60 Antimony Potassium Tartrate.tw.

61 Antimony Sodium Gluconate.tw. =

62 Hycanthone.tw.

63 Lucanthone.tw.

64 Niridazole.tw.

65 Oxamniquine.tw.

66 Praziquantel/

67 Trichlorfon/

68 metrifonate.tw.

69 Artemisinins/

70 (artesunate or artemether).tw.

71 or/34‐72

72 (deworm* or de‐worm*).tw.

73 exp Anthelmintics/ or (anthelmint* or antihelmint*).tw.

74 72 or 73

75 Pregnant Women/ or Pregnancy/ or Pregnancy Complications, Parasitic/ pregnant wom*n .tw.

76 32 and 71

77 74 or 76

78 75 and 77

Database: Embase Classic+Embase

1 whipworm*.tw.

2 whip worm*.tw.

3 hookworm*.tw.

4 hookworm*.tw.

5 hook worm*.tw.

6 roundworm*.tw.

7 round worm*.tw.

8 pinworm*.tw.

9 pin worm*.tw.

10 flukes.tw.

11 geohelminth*.tw.

12 ancylostoma.tw.

13 Necator*.tw.

14 Ascaris.tw.

15 Ascaridida.tw.

16 Ancylostoma.tw.

17 Necator americanus.tw.

18 Enterobius.tw.

19 Oxyuroidea.tw.

20 Oxyurida.tw.

21 Trichuris.tw.

22 Trichuroidea.tw.

23 Capillaria.tw.

24 Trichinella.tw.

25 Strongyloid*.tw.

26 Oesophagostomum.tw.

27 Oesophagostomiasis.tw.

28 Acanthocephala.tw.

29 Adenophorea.tw.

30 Enoplida.tw.

31 Secernentea.tw.

32 Ascaridida.tw.

33 Rhabditida.tw.

34 Cestoda.tw.

35 Trematod*.tw.

36 Turbellaria.tw.

37 Platyhelminth*.tw.

38 Rotifera.tw.

39 trichuriasis.tw.

40 ascariasis.tw.

41 trichinellosis.tw.

42 Trichostrongyloidiasis.tw.

43 ancylostomiasis.tw.

44 enterobiasis.tw.

45 cestode*.tw.

46 trematode*.tw.

47 ascarid*.tw.

48 schistosomiasis.tw.

49 Schistosoma*.tw.

50 or/1‐49

51 Albendazole/

52 Mebendazole/

53 exp Piperazines/

54 Levamisole/

55 exp Pyrantel/

56 Ivermectin/

57 exp Anthelmintics/

58 Ivermectin.tw.

59 Albendazole.tw.

60 Mebendazole.tw.

61 Piperazine*.tw.

62 Levamisole.tw.

63 pyrantel.tw.

64 tiabendazole.tw.

65 anthelmint*.tw.

66 *Antiplatyhelmintic Agents/

67 Anticestodal.tw.

68 Antiplatyhelmintic.tw.

69 Anti‐platyhelmintic.tw.

70 Albendazole.tw.

71 Dichlorophen.tw.

72 Niclosamide.tw.

73 Bithionol.tw.

74 Diamfenetide.tw.

75 Nitroxinil.tw.

76 Oxyclozanide.tw.

77 Rafoxanide.tw.

78 Schistosomicide*.tw.

79 Antimony Potassium Tartrate.tw.

80 Antimony Sodium Gluconate.tw.

81 Hycanthone.tw.

82 Lucanthone.tw.

83 Niridazole.tw.

84 Oxamniquine.tw.

85 or/51‐84

86 (deworm* or de‐worm*).tw.

87 anthelmint*.tw.

88 anthelmintic/

89 or/86‐88

90 pregnant wom*n .tw.

91 (woman or women).tw.

92 pregnan*.tw.

93 or/90‐92

94 50 and 85

95 94 or 89

96 95 and 93

Cochrane Library – CDSR, DARE, CENTRAL, EED, HTA

ID Search

#1 helmint*:ti,ab,kw (Word variations have been searched)

#2 Ancylostoma duodenale

#3 Necator americanus

#4 Ascaris

#5 Enterobius vermicularis

#6 trichuris

#7 Strongyloid*

#8 hookworm*

#9 roundworm*

#10 pinworm*

#11 whipworm*

#12 schistosomiasis

#13 Schistosoma

#14 #1 or #2 or #3 or #4 or #5 or #6 or #7 or #8 or #9 or #10 or #11 or #13

#15 albendazole

#16 mebendazole

#17 piperazine

#18 levamisole

#19 pyrantel

#20 tiabendazole

#21 deworm*:ti,ab or de‐worm*:ti,ab

#22 #15 or #16 or #17 or #18 or #19 or #20 or #21

#23 #21 or #22

#24 #23 and #14

#25 deworm

#26 de‐worm

#27 deworming

#28 de‐worming

#29 anthelmint*

#30 anthelmintic

#31 #25 or #26 or #27 or #28 or #29 or #30

#32 #24 or #31
